# Antimicrobial resistance among GLASS pathogens in conflict and non-conflict affected settings in the Middle East: a systematic review

**DOI:** 10.1186/s12879-020-05503-8

**Published:** 2020-12-09

**Authors:** Claudia Truppa, Mahmoud N. Abo-Shehada

**Affiliations:** 1 International Committee of the Red Cross, Geneva, Switzerland; 2 Faculty of Epidemiology and Population Health, London School of Hygiene and Tropical Medicine, University of London, London, UK

**Keywords:** Antibiotic resistance, Antimicrobial resistance, Middle East, Conflict

## Abstract

**Background:**

In spite of the evident general negative effects of armed conflict on countries’ health systems and populations’ health outcomes, little is known about similar impacts of conflicts on the spread of antimicrobial resistances (AMR). This review was to address this evidence gap and describe:

1. Patterns of AMR in the Middle East (ME) and resistance profiles of pathogens included in the Global AMR Surveillance System (GLASS) supported by the World Health Organization;
2. Differences in proportions of AMR isolates between conflict and non-conflict countries.

**Methods:**

A systematic literature review was conducted following PRISMA guidelines and searching five electronic databases. Subject heading and free text were searched for “antimicrobial resistances” and “Middle East”, to identify observational studies on AMR published from January 2011 to June 2018. Data were extracted from included articles on a predefined set of variables. Percentages of AMR were analysed as median and interquartile ranges. Risk of bias was assessed using the Newcastle-Ottawa Scale.

**Results:**

A total of 132 articles met the inclusion criteria. Included studies showed heterogeneity in study design, laboratory methods and standards for interpretation of results, and an overall high risk of bias. Main findings were the following:

1. High proportions of carbapenem resistance in *Acinetobacter spp.* (median 74.2%), and both carbapenem resistance (median 8.1 and 15.4% for *E. coli* and *K. pneumoniae* respectively) and ESBL-production (median 32.3 and 27.9% for *E. coli* and *K. pneumoniae* respectively) amongst *Enterobacteriaceae*. *S. aureus* isolates showed a median methicillin resistance percentage of 45.1%, while vancomycin resistance was almost absent. A median of 50% of the strains of *S. pneumoniae* showed non-susceptibility to penicillin.
2. Similar trends were observed in conflict and non-conflict affected countries.

**Conclusions:**

There is a lack of standardization in the methodological approach to AMR research in the Middle East. The proportion of antibiotic resistances among specific GLASS pathogens is high, particularly among *Acinetobacter spp.*

**Supplementary Information:**

**Supplementary information** accompanies this paper at 10.1186/s12879-020-05503-8.

## Background

Antimicrobial resistance (AMR), and more specifically antibiotic resistance (ABR), have become in the past two decades a globally rising concern. Since 2001, the World Health Organization (WHO) called for joining efforts in addressing the growing threat of AMR [1], following the pioneer example of the Pan-American Health Organization in establishing a surveillance network at regional level [2]. However, it was not until 2009–2010 that other regional action plans and surveillance systems were put in place, with such actions taking place initially in high income countries [3, 4].

In 2014, the World Health Assembly (WHA) issued resolution WHA67.25, calling for the development of a global action plan against AMR [5]. WHO took the lead of such process, publishing in 2015 a Global Action Plan on AMR [6], and establishing the Global AMR Surveillance System (GLASS) in order to ensure evidence base and integrated data collection and surveillance across countries [7].

The global call against AMR has found echo in many regions since then, with the progressive establishment of harmonized surveillance systems in Eastern Europe and Central Asia [8], and thourough situation analysis conducted in the South East Asia WHO Region [9].

Critically, many Low and Middle-Income Countries, particularly in Africa and the Middle East, have not yet fully established similar regional networks. Recently, systematic reviews on AMR have been conducted in the West Pacific Region [10] and in Africa, showing high prevalence of resistances to the most commonly used antibiotics, and highlighting the critical evidence gap from many countries of the African Region [11–13]. No similar attempts to collate evidence available in the Middle East have been performed so far. Recent studies conducted in high income countries on AMR in refugees, including refugees from the Middle East, have led to hypothesize an association between refugee status and risk of AMR transmission. However, such studies have failed to provide any evidence on whether the AMR was acquired in the home country, during the journey or in the host countries [14, 15].

The lack of evidence on the magnitude of AMR in the Middle East is not surprising, considering the well-known health effects of the peculiar geopolitical dynamics of this area of the world: in fact the Middle East has witnessed over the past decades a sustained social and political turmoil, with more than half of the countries members of the Arab League being theatres of protracted armed conflict [16]. Since 2011 the situation has further escalated, with the Arab Spring and the conflicts in Syria, Yemen, and Mosul in Iraq [17].

Middle Eastern health systems in countries witnessing protracted armed conflict or carrying the burden of populations fleeing from them, have been progressively weakened, have seen their basic services disrupted, their health infrastructures damaged or destroyed, and a substantial proportion of their workforce either fleeing or being killed [18, 19]. Armed conflicts have been already shown to have, in virtue of the above mentioned effects on the health system, a tremendous impact on the health outcomes of the populations affected, in terms of increased morbidity, mortality and disability [20–24].

Armed conflict and political instability in the Middle East constitute obstacles for the implementation of health policies and global action plans, including those related to proper AMR surveillance and stewardship. However, despite the fact that emergence of drug resistance has already been described as one of the potential effects of armed conflict since over more than one decade [25], no formal and systematized evidence has been gathered so far. Sporadic evidence has recently highlighted that victims of armed conflict might be at higher risk of harbouring and/or being infected by multidrug resistant (MDR) bacteria [26, 27], but overall AMR in the Middle East remains poorly documented, and no study conducted so far has ever attempted exploring the magnitude of the problem of AMR in conflict-affected settings in the region.

There are multiple pathways through which armed conflict can contribute to the creation and spread of AMR in the Middle East: disruption of healthcare systems; delayed access to healthcare; increased nosocomial transmission of resistant pathogens; increased community transmission of resistant pathogens in settings as refugee camps; poor antimicrobial stewardship in the countries affected and in humanitarian interventions; etc. [16, 20, 23, 28, 29].

This is the first review addressing explicitly the evidence gap on AMR in the Middle East, and the first one in attempting to explore specifically the hypothesized association between conflict and AMR.

## Methods

The main objectives of this review are:

1. To describe the patterns of AMR in the Middle East and the specific resistance profiles of pathogens listed in the WHO-supported GLASS; and
2. To identify any differences in AMR profiles in populations in conflict affected settings as compared to populations in non-conflict affected settings.

### Search strategy

A systematic literature review was conducted following the Preferred Reporting Items for Systematic Reviews and Meta-Analysis (PRISMA) guidelines [30] (see Additional file 1: Appendix 1) and searching the following electronic databases: PubMed, MEDLINE, Embase, Global Health, CINAHL Plus, IMEMR (Index Medicus for the Eastern Mediterranean Region), *Médecins Sans Frontières* (MSF) and the International Committee of the Red Cross websites and the reference lists of the selected articles.

The search strategy included subject heading searches and free text searches for the following keywords: “*antimicrobial resistances*”, “*antibiotic resistances*”, “*antimicrobial susceptibility*” and, the name of countries in the review, in addition to all relevant synonyms. The detailed search strategy is reported in Additional file 1: Appendix 2.

**Fig. 1:**
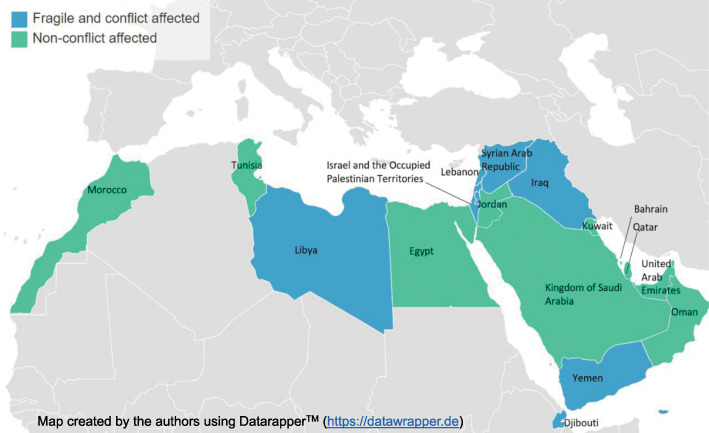
Map of countries included in this systematic review on AMR and conflict in the Middle East (the identification as fragile and conflict affected and non-conflict affected is based on the World Bank classification, 2011–2018)

A standard definition of “Middle East” is difficult to retrieve [31], and several sources include different countries under such title. Within the scope of this review, Middle East has been defined as the geopolitical area comprising all countries part of both the WHO Eastern Mediterranean Region (EMR) [32] and the World Bank Middle East and North Africa Region [33], and members of the League of Arab States [34], in order to include settings sharing the same language and whose political and social dynamics are strictly intertwined. As a result, eighteen countries are included in this study (Fig. 1).

Search items were imported in the reference manager software EndNote version X8.2. After removal of duplicates, screening of titles and abstracts was performed, and articles selected for full text screening were obtained. Full text was obtained either using the university library services or by directly contacting the author(s). Articles for which no access was obtained from university library services or if no reply was received within three weeks of the request to the author(s), were excluded from the review.

### Selection criteria

Observational studies included in this review were cohort and cross-sectional studies describing the proportion, prevalence or incidence of AMR in Arab Middle Eastern civilian populations of both sexes and all ages (neonates: aged less than 28 days; infants: aged 28 days to 1 year; children: more than 1 year to 17 years of age; adults: aged 18 years or more), living in the above-named countries. Any other study, regardless of study design, which clearly specified the total number of isolates as denominator and the resistance profiles of individual pathogens, was also included, as suggested by Omulo et al. in a recent review on AMR research [13].

### Eligibility criteria

Peer reviewed papers published since 2011 (beginning of the Arab Spring and the Syrian crisis) until mid-2018 in Arabic, English, French, Italian, Portuguese and Spanish were sought, on bacterial resistances in civilian populations living in the Middle East, reporting on data collected after the beginning of 2011 on AMR in the eight GLASS pathogens.

Papers dealing with non-Arab migrants in the Middle East, or military personnel deployed from non-Middle Eastern countries for combat operations in the Middle East, were excluded, considering that AMR carriage and/or infection profiles of these populations could not be representative of the patterns of AMR in the area under study, as they could harbour AMR contracted in their home countries. Similarly, papers on AMR profiles in Arab Middle Eastern refugees in high income countries were excluded, as it would be impossible to ascertain whether the AMR profile was acquired in the home countries, during the migration journey, or in the host countries.

Exclusion criteria adopted were applied hierarchically in the following order: peer reviewed papers describing studies conducted outside the above defined geographical area of interest; reporting AMR data collected before 2011 or on AMR in other pathogens not included in GLASS; articles describing veterinary or environmental studies; studies on AMR patterns in military personnel deployed from non-Middle Eastern countries in the Middle East, or collected from non-Middle Eastern populations residing in any of the included countries; articles characterizing exclusively clusters of AMR bacteria and without information on the total of isolated microorganisms regardless of resistance profiles; intervention and analytical studies recruiting participants or specimens on the basis of a specific ABR profile; articles not available in full text in any of the above mentioned languages; correspondence, comments, case reports and case series.

#### Types of exposures

Countries were classified as “conflict-affected” if they were included in the World Bank list of “fragile and conflict-affected situations” [35]. According to the list, the following eight countries included in the review were defined as conflict-affected: Djibouti, Iraq, Israel and the Occupied Palestinian Territories (West Bank and Gaza), Lebanon, Libya, Syrian Arab Republic, and Yemen (Fig. 1), and the rest of studied countries were non-conflict affected.

#### Types of outcome measures

Primary outcome measures extracted were: incidence risk, prevalence, and proportion of AMR isolates.

### Data collection process

A data extraction form was designed including: 1) Article information: first author, year of publication, year(s) of data collection, country in which the study was conducted, classification of the country as conflict-affected or non-conflict-affected; 2) Study information: type of study, study setting (community, primary care or hospital), study population, classification of the study population as conflict-affected or non-conflict-affected, sample size, age group(s), number of samples (or non-duplicate isolates, if available) collected, clinical syndrome(s) under study, primary outcome measure(s); 3) Microbiological information: pathogen(s) isolated, laboratory methodology employed for identification and antibiotic susceptibility testing (AST), criteria used for interpretation of resistances, AMR profile(s).

The data extraction form was designed based on the recommendations formulated by Omulo in her 2015 work [13], in order to promote comparisons of results across different low and middle income settings, as these were also adopted in a recent systematic review on AMR in Africa [11]. For the definition of the AMR profile, the total number of isolates included in the study was extracted, along with the number of non-susceptible isolates for all the antibiotics tested. The main AMR profiles were defined as those identified by WHO as “priority pathogens” for the public health significance they have; in the specific case of GLASS pathogens, these include: carbapenem-resistant *Acinetobacter baumannii*, carbapenem-resistant and Extended Spectrum Beta Lactamase (ESBL)-producing *Enterobacteriaceae* (*E. coli* and *K. pneumoniae*), third generation cephalosporin-resistant *N. gonorrhoeae*, methicillin-resistant and vancomycin-resistant *S. aureus* (MRSA and VRSA respectively), fluoroquinolone-resistant *Salmonella spp.* and *Shigella spp.*, and penicillin non-susceptible *S. pneumoniae* [36].

For articles presenting data on multiple pathogens, data were collected exclusively on the resistance profiles of the GLASS pathogens. Where resistance profiles were indicated exclusively for MDR pathogens, data were extracted only on primary resistance profiles demonstrated in the initial total sample included in the study. Intermediate susceptibility, where reported, was consolidated with susceptibility, as per experts’ recommendations at the time of writing, and formalized later in 2019 in the EUCAST guidance [37].

The data extraction form is detailed in Additional file 1: Appendix 3. Data were successively extracted in Microsoft Excel 2016 for analysis. A quantitative analysis was performed on 116 articles reporting on the above mentioned specific antibiotic resistances of public health significance, which consisted of the calculation of resistance median percentages and interquartile range (IQR).

Publication bias was assessed designing funnel plots for articles on one pathogen isolated from specific collection samples, where the number of studies was sufficient to allow the analysis, and performing Egger’s regression test for statistical evidence of publication bias, using Stata™ software version 14.2.

### Assessment of risk of bias

An initial screening tool for overall quality appraisal was used. A subsequent in-depth assessment of risk of bias was performed adopting a modified version of the Newcastle-Ottawa Scale for cohort studies [38], and an adapted version to cross-sectional studies (both the screening tool and the modified scales are available in Additional file 1: Appendix 3), as it was recommended at the time of writing by the Cochrane Collaboration for the assessment of risk of bias in non-randomized studies [39].

The following categories were assessed: selection of participants or specimens, comparability of study groups, and outcome or exposure assessment according to the study type. Within each category, a variable number between two and three items was assessed, and colour coded according to the extent to which the study demonstrated a good (green), moderate, incomplete or doubtful (yellow), or poor (red) abidance to the epidemiological standards and good practices for the study design chosen.

Articles were classified as at high, moderate or low risk of bias according to the following criteria: a) High risk: poor epidemiological design in the three categories; b) Moderate risk: good epidemiological design in two categories or acceptable design in the three categories; and c) Low risk: good epidemiological design across all categories.

## Results

### Study selection and characteristics

**Fig. 2:**
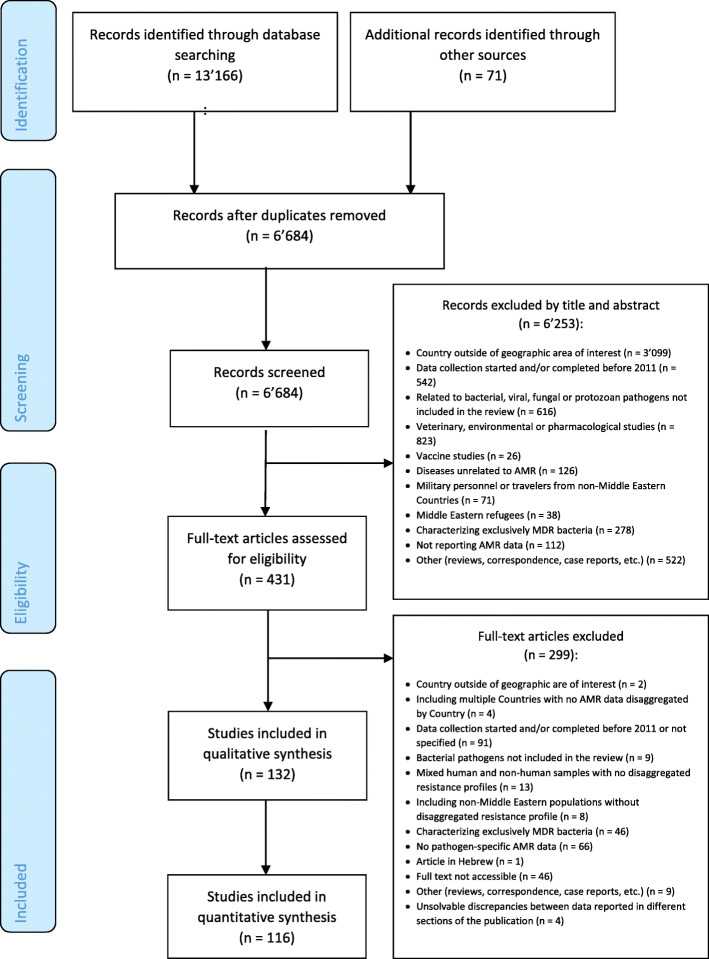
PRISMA flow diagram of the selection of articles for this review

A total of 13′166 records were identified; 4′228 items in Embase, 2′223 in Medline, 3′541 in Global Health, 2′603 items in PubMed, and 571 in CINAHL Plus and additional 71 records were identified, 68 items in IMEMR and 3 items in the MSF Field Research Website [40]. After removing duplicates, titles and abstracts of 6′684 records were screened, of which only 431 were eligible for full text screening. Of the 431 articles read in full text, 132 met the inclusion criteria (Fig. 2) and were included.

Table 1 describes the study characteristics. All the articles selected for inclusion in the study were in English except one study that was in French [100]. More than half of included studies were conducted in only three countries: Egypt (26.5%), KSA (18.9%), and Iraq (18.2%). In all the other countries included in the review only between one and less than ten articles were included, and two countries, Djibouti and Qatar, had no included article.

**Table 1:** Summary of the characteristics of the 132 articles included in the systematic review on antimicrobial resistances among GLASS pathogens in conflict and non-conflict affected settings the Middle East

Study characteristics	Number (%)	References
Year of publication		
2012	2 (1.5)	[41, 42]
2013	11 (8.3)	[43–51]
2014	17 (12.9)	[54–68]
2015	34 (25.8)	[71–102]
2016	28 (21.2)	[105–130]
2017	28 (21.2)	[133–158]
2018	12 (9.1)	[161–170]
Year of completion of data collection		
2011	14 (10.6)	[41, 48, 49, 54, 58, 66, 74, 78, 85, 86]
2012	13 (9.8)	[51, 56, 59, 60, 62, 63, 68, 70, 71, 105, 109]
2013	31 (23.5)	[46, 47, 50, 55, 61, 64, 67, 69, 72, 73, 75, 80, 81, 87–89, 91, 92, 94, 99–101, 113, 120, 123, 127, 132, 145, 153, 155, 159]
2014	26 (19.7)	[57, 65, 76, 79, 83, 84, 90, 93, 95–98, 104, 114, 116, 124, 125, 129, 130, 135, 149, 154, 157, 158, 160, 167]
2015	31 (23.5)	[77, 82, 102, 103, 106–108, 110–112, 115, 117–119, 121, 122, 126, 128, 131, 134, 136, 140, 142–144, 147, 148, 150, 151, 166, 171]
2016	14 (10.6)	[133, 138, 139, 141, 152, 156, 161, 163–165, 168–170, 172]
2017	3 (2.3)	[137, 146, 162]
Country of publication		
Non-conflict affected	**81 (61.4)**	
Bahrain	0 (0)	NA
Egypt	35 (26.5)	[42, 47, 52, 61, 65, 68, 70–73, 87, 89, 91, 93, 94, 98, 99, 103, 105, 120, 121, 125, 131, 133, 134, 138, 139, 142, 145, 146, 148, 150, 166, 168, 172]
Jordan	8 (6.1)	[49, 57, 88, 119, 132, 161, 163, 169]
Kingdom of Saudi Arabia (KSA)	25 (18.9)	[41, 46, 51, 62, 69, 76, 77, 80, 81, 83, 85, 90, 92, 102, 107, 108, 111, 115–117, 135, 136, 154, 164, 170]
Kuwait	1 (0.8)	[130]
Morocco	7 (5.3)	[63, 64, 86, 100, 143, 153, 155]
Oman	0 (0)	NA
Qatar	0 (0)	NA
Tunisia	1 (0.8)	[152]
United Arab Emirates (UAE)	2 (1.5)	[60, 165]
Multiple countries: Egypt and KSA	2 (1.5)	[74, 97]
Fragile and conflict affected	**50 (37.9)**	
Djibouti	0 (0)	NA
Iraq	24 (18.2)	[53, 56, 58, 67, 78, 79, 82, 84, 95, 96, 109, 110, 112, 114, 118, 126, 129, 137, 140, 151, 156, 158, 162, 171]
Israel and the Occupied Palestinian Territories	7 (5.3)	[43, 44, 50, 54, 113, 144, 147]
Lebanon	8 (6.1)	[66, 101, 106, 123, 124, 149, 157, 167]
Libya	6 (4.5)	[48, 59, 75, 104, 159, 160]
Syrian Arab Republic	4 (3.0)	[45, 55, 128, 141]
Yemen	1 (0.8)	[122]
Mixed conflict and non-conflict affected	**1 (0.7)**	
Bahrain, Oman, UAE and Lebanon	1 (0.7)	[127]
Study design		
Cross-sectional	16 (12.1)	[45, 50, 74, 78, 91, 93, 95, 96, 102, 107, 122, 138, 144, 146, 163, 164]
Case-control	2 (1.5)	[57, 165]
Cohort	2 (1.5)	[131, 161]
Surveillance	9 (6.8)	[52, 109, 116, 123, 127, 135, 149, 159, 160]
Not specified	103 (78.1)	[41, 42, 46–49, 51, 53–56, 58–73, 75–77, 80–90, 92, 94, 97–101, 103–106, 108, 110–115, 117–121, 124–126, 128–130, 132–134, 136, 137, 139–143, 145, 147, 148, 150–158, 162, 166–172]
Study setting		
Hospital	66 (50.0)	[46, 51–53, 55, 56, 59, 61, 62, 64, 65, 67, 68, 71, 72, 75–77, 81–84, 86, 87, 89–93, 96, 99–101, 103, 104, 107–109, 111, 114, 115, 119, 121, 131–133, 136, 142, 144, 146–148, 150, 155–158, 161, 162, 164, 168, 170, 172]
Laboratory	55 (41.7)	[41, 45, 49, 54, 58, 60, 63, 66, 69, 70, 73, 78–80, 85, 88, 94, 97, 98, 106, 110, 112, 113, 116–118, 120, 123–129, 134, 135, 137–141, 143, 145, 149, 151–154, 159, 160, 165–167, 169, 171]
Primary care	4 (3.0)	[50, 74, 122, 130]
Community	3 (2.3)	[95, 102, 163]
Mixed	4 (4.0)	[43, 44, 57, 105]
Source of data		
Inpatients	66 (50.0)	[41, 42, 46–48, 51–56, 60–63, 65, 68, 69, 71–73, 82, 83, 85, 86, 89–92, 96, 99–101, 103, 104, 106–111, 115–118, 121, 124, 126, 131, 133, 134, 136, 138–140, 147, 150, 151, 153, 156, 159, 161, 162, 167, 168, 172]
Outpatients	12 (9.1)	[50, 57, 59, 74, 77, 84, 97, 114, 122, 130, 132, 155]
Healthcare workers	5 (3.8)	[81, 87, 144, 148, 170]
Students	4 (3.0)	[44, 95, 102, 163]
*Of which medical students*	*2 (1.5)*	[102, 163]
Mixed	17 (12.9)	[43, 45, 66, 70, 75, 76, 105, 119, 137, 141, 145, 146, 154, 164–166, 169]
Not specified	28 (21.2)	[49, 58, 64, 67, 78–80, 88, 93, 94, 98, 112, 113, 120, 123, 125, 127–129, 135, 142, 143, 149, 152, 157, 158, 160, 171]
Age groups*		
Neonates	6 (4.5)	[55, 71, 92, 119, 133, 156]
Infants	15 (11.4)	[55, 64, 86, 93, 101, 103, 108, 109, 114, 119, 127, 132, 145, 146, 156]
Children	33 (25.0)	[50, 55, 62, 64, 67, 77, 79, 84, 86, 90, 93, 95, 100, 101, 103, 107–109, 114, 115, 122, 127, 132, 136, 139, 145, 146, 153, 155, 156, 159, 169, 172]
Adults	44 (33.3)	[53, 56, 61, 62, 65, 76, 81, 84, 86, 87, 89, 90, 95, 99, 101, 102, 107–109, 111, 114, 115, 130, 131, 136, 144, 145, 147, 148, 153, 155, 159, 161–163, 168–170, 172]
All age groups	13 (9.8)	[41, 43, 45, 46, 51, 52, 68, 75, 107, 127, 142, 152, 164]
Not specified	58 (43.9)	[49, 54, 57–60, 63, 66, 69, 70, 72–74, 78, 80, 82, 83, 85, 88, 91, 94, 96–98, 104–106, 110, 112, 113, 117, 118, 120, 121, 123–126, 128, 129, 134, 135, 137, 140, 141, 143, 149–151, 154, 157, 158, 160, 165–167, 171]
Gender		
Male	1 (0.7)	[48]
Female	0 (0)	NA
Both	67 (50.8)	[41–44, 49–53, 61, 62, 65, 66, 71, 74–77, 81, 86, 88–90, 95, 98–102, 107–109, 111, 114–116, 119, 122, 127, 130–133, 136, 141, 142, 145–148, 152–157, 160–165, 168, 169, 172]
Not specified	64 (48.5)	[47, 54–60, 63, 64, 67–70, 72, 73, 78–80, 82–85, 87, 91–94, 96, 97, 103–106, 110, 112, 113, 117, 118, 120, 121, 123–126, 128, 129, 134, 135, 137–140, 143, 144, 149–151, 158, 159, 166, 167, 170, 171]
Clinical syndrome		
Colonization	21 (15.9)	[41, 44, 50, 51, 57, 74, 77, 81, 83, 87, 92, 93, 95, 102, 111, 119, 132, 144, 148, 163, 170]
Urinary tract infections	20 (15.2)	[45, 49, 54, 59, 75, 84, 100, 107, 109, 114, 130, 146, 147, 154, 155, 159, 164–166, 169]
Skin and soft tissue infections	10 (7.6)	[42, 48, 61, 82, 96, 121, 125, 126, 140, 161]
*Of which war-related*	*2 (1.5)*	[48, 161]
Bloodstream infections	10 (7.6)	[65, 71, 72, 103, 115, 133, 137, 150, 153, 172]
Respiratory tract infections	6 (4.5)	[62, 64, 89, 99, 122, 162]
Gastroenteritis	5 (3.8)	[58, 67, 79, 98, 149]
Meningitis	2 (1.5)	[134, 156]
Keratitis	1 (0.7)	[142]
Miscellanea	43 (32.6)	[43, 46, 47, 52, 53, 55, 56, 60, 63, 66, 68–70, 73, 76, 78, 86, 88, 90, 91, 94, 101, 104, 106, 108, 112, 116–118, 120, 124, 127, 129, 131, 136, 138, 139, 141, 145, 151, 152, 157, 168]
Type of specimen		
Urine	20 (15.2)	[45, 49, 54, 59, 75, 84, 100, 107, 109, 114, 130, 146, 147, 154, 155, 159, 164–166, 169]
Nasal/nasopharyngeal swab	18 (13.6)	[41, 44, 50, 51, 56, 57, 74, 77, 81, 93, 95, 102, 111, 132, 144, 148, 163, 170]
Blood	10 (7.6)	[65, 71, 72, 103, 115, 133, 137, 150, 153, 172]
Tissue swab/biopsy	9 (6.8)	[42, 48, 61, 82, 121, 125, 126, 140, 161]
Faeces	8 (6.1)	[58, 67, 79, 87, 92, 98, 119, 149]
Sputum or other respiratory	4 (3.0)	[62, 89, 99, 162]
Other	7 (5.3)	[64, 83, 104, 122, 134, 142, 156]
Miscellanea	52 (39.4)	[43, 46, 47, 52, 53, 55, 60, 63, 66, 68–70, 73, 76, 78, 86, 88, 90, 91, 94, 97, 101, 105, 106, 108, 110, 112, 113, 116–118, 120, 123, 124, 127–129, 131, 135, 136, 138, 139, 141, 143, 145, 151, 152, 157, 158, 167, 168, 171]
Not specified	4 (3.0)	[80, 85, 96, 160]
Laboratory methods		
Identification of microorganism		
Morphology	53 (40.2)	[41–44, 47, 48, 50, 51, 54–58, 61–63, 66, 67, 73, 74, 78, 79, 81, 87, 91, 93–96, 99, 100, 102, 105, 109, 111, 113, 114, 119, 122, 125, 127–129, 132, 136, 142, 144, 146, 148, 150, 152, 156, 169]
VITEK™	21 (15.9)	[46, 72, 75, 76, 80, 82–85, 88, 101, 106, 108, 117, 118, 140, 151, 154, 158, 162, 171]
API	15 (11.4)	[45, 59, 68, 70, 86, 90, 124, 131, 143, 145, 149, 153, 155, 157, 167]
Others	12 (9.1)	[69, 77, 104, 107, 115, 116, 130, 133, 159, 160, 164, 170]
Multiple	25 (17.4)	[53, 60, 64, 65, 92, 97, 98, 103, 110, 112, 120, 121, 123, 126, 135, 137–139, 147, 161, 163, 165, 166, 168, 172]
Not specified	6 (4.5)	[49, 52, 71, 89, 134, 141]
Antibiotic susceptibility testing		
Disk diffusion	64 (48.5)	[42, 43, 47–52, 54, 57–59, 64, 65, 67, 68, 70, 71, 74, 76, 79, 81, 82, 86, 87, 91, 94, 96, 98–100, 105, 106, 109, 110, 112, 113, 119–121, 124–126, 128, 130, 136, 137, 141–146, 148, 149, 152, 153, 155, 156, 162, 166, 169]
VITEK™	12 (9.1)	[83, 88, 92, 108, 117, 118, 135, 151, 154, 158, 168, 171]
PCR	7 (5.3)	[55, 62, 66, 78, 101, 102, 129]
E-test™	4 (3.0)	[97, 127, 131, 160]
MIC	4 (3.0)	[46, 53, 90, 134]
Others	3 (2.3)	[77, 104, 115]
Multiple	35 (26.5)	[41, 56, 60, 61, 63, 69, 72, 73, 75, 80, 84, 85, 93, 95, 103, 107, 111, 114, 116, 123, 132, 138–140, 147, 150, 157, 159, 161, 163–165, 167, 170, 172]
Not specified	3 (2.3)	[89, 122, 133]
Standard for interpretation of results		
CLSI	103 (78.0)	[41–46, 50–52, 54–57, 59–61, 63–67, 69–71, 74–76, 79–82, 84, 86, 88, 90–92, 94–96, 98–100, 102, 103, 105–121, 124–126, 130, 132–134, 136–150, 155–159, 161–169, 172]
BSAC	3 (2.3)	[85, 97, 122]
EUCAST	2 (1.5)	[72, 160]
CA-SFM	2 (1.5)	[152, 153]
Multiple methods	4 (3.0)	[53, 68, 124, 127]
Not specified	18 (13.6)	[49, 58, 62, 73, 77, 78, 87, 89, 101, 104, 128, 129, 131, 135, 151, 154, 170, 171]

* Articles can report data on more than one age group, and therefore the sum of all age groups can differ from the total number of articles

*GLASS* Global Antimicrobial Resistance Surveillance System

*NA* Not Applicable

*API* Analytical Profile Index

*PCR* Polymerase Chain Reaction

*MIC* Minimum inhibitory concentration

*CLSI* Clinical & Laboratory Standards Institute

*BSAC* British Society for Antimicrobial Chemotherapy

*EUCAST* European Committee on Antimicrobial Susceptibility Testing

*CA-SFM Comité de l’Antibiogramme de la Societé Française de Microbiologie*

A higher proportion (61.4%) of articles were from stable countries and 51 articles, described AMR in fragile and conflict-affected countries: almost half of these were published in Iraq (Table 1).

Out of the 132 articles, 107 did not specify the nationality of the individuals from which the specimens were obtained. No assumption was made that a study conducted in one country would describe exclusively the population native of the same country, in virtue of the intense migration patterns from outside and within the Middle East [173, 174].

The vast majority of studies included were conducted in hospital and hospital laboratory settings (50.0 and 41.7% respectively). Community and primary health care settings where largely underrepresented (5.3%). Inpatients constituted the source of half of the data (50.0%) compared to outpatients. The source of microbiological data was not specified in 21.2% of studies.

Similarly, almost half (48.5%) of the included articles did not specify the age groups and/or genders included in the study population. When specified, adults were the most commonly studied population (33.3% of the included papers), followed by children (25.0%). No studies from conflict-affected settings, however, presented data on children. Only one paper reported results from a study population entirely represented by men [48].

Only five articles were investigating AMR in immunosuppressed populations, represented either by HIV-infected patients [59], subjects who underwent liver transplantation [47], or patients affected by different types of malignancies [91, 115, 172]. Of these, only one was conducted in a conflict affected setting [59].

Only two articles presented ABR in diabetic patients [42, 61]: these were both from Egypt, and referred to skin and soft tissue infections.

### Appraisal of risk of bias

The detailed summary of the assessment of risk of bias performed is available in Additional file 1: Appendix 3 and 4.

All studies included in this review were screened for the completeness of information provided including; the study design, research question and objectives, and description of the findings in terms of person, place and time, along with the justification of the sample size included and the provision of a measure of random variation of the presented results.

The study design was clearly stated in 29 out of the 132 articles included (Table 1). Although the study question was rarely focused in terms of population of interest, geographic area and time span of observation (18.9%, 25/132), the study objectives were detailed in the majority of papers (77.3%, 102/132). Time period of observation and geographical area of interest were clearly specified in 96.2% (127/132) of articles, while detailed demographic information on the study population, at least in terms of age groups and gender included, were available for 32.6% (43/132).

Only one study reported sample size calculation and employed representative population although not powered for the prevalence detected nor for the comparison between the sub-groups performed [95]. Measures of sampling variation in the results were reported by only four studies [95, 161, 165, 168].

Statistical methods were in general poorly detailed, and mostly summarized in terms of descriptive statistics. Only three studies described the use of multivariate analysis – usually logistic regression – to control for confounding [45, 147, 168]. None of the studies took missing data into account in the analysis nor in the discussion, and similarly limitations and potential sources of bias were never mentioned in the discussion, with the exception of one surveillance study performed in Lebanon [123].

A substantial number of articles mentioned exclusively clinical suspicion from the attending physician as diagnostic criteria and rarely a clear case definition was detailed for the clinical syndrome under study. No clear sources for patient’s socio-demographic data were descripted.

In general, cross-sectional studies presented major flaws in terms of sample selection, statistical methods employed (not described in the majority of cases), and possibility of misclassification of both exposures and outcome under study.

The cohort studies included in the review [52, 131, 161] showed overall lower risk of bias compared to other study designs. Two out of the three studies [52, 131] lacked detailed description of the statistical methods employed, in particular if and how control for confounding was performed and how losses to follow up were dealt with. Remarkably, one of the cohort studies included was conducted in a non-conflict affected country, but describing findings on a conflict-affected population, as it investigated AMR in war wounded civilian Syrian patients admitted to a Jordanian hospital [161].

The two case-control studies included [57, 165] poorly described the inclusion criteria for the cases and did not clearly detail the recruitment of controls. Data extracted from these two studies were not included in the quantitative analysis.

### Synthesis of results

Patterns of ABR in the Middle East and differences between conflict-affected and non-conflict-affected countries.

The most commonly described pathogens were, in order of frequency, *E. coli* (40.9% of the included studies, 54/132), *S. aureus* (34.1%, 45/132), *Acinetobacter* spp. (29.5%, 39/132), and *K. pneumoniae* (25.8%, 34/132), both in conflict and non-conflict affected settings. Other bacteria were less represented in this review, and no study investigated AMR in *N. gonorrhoeae.*

Antibiotic susceptibility testing (AST) was performed for a total of 73 different antibiotics, with substantial inhomogeneity across studies for each pathogen. Due to the substantial heterogeneity of methodologies adopted, it was not possible to perform a meta-analysis as quantitative synthesis of the results from the standpoint of AMR profile of the studied pathogens: such heterogeneity was due to the differences in terms of study settings, study populations included in the studies, laboratory methodologies employed to appraise susceptibility to the different antibiotics, and clinical conditions investigated. In addition to this substantial variability, there was a significant proportion of studies with missing information on the above mentioned categories, along with an overall high risk of selection bias, misclassification of the exposure categories, and lack of control for confounding. However, in Table 2 median percentages and IQR of specific AMR profiles are reported for selected GLASS pathogens, focusing on the most frequently reported ones.

**Table 2:** Proportion of antibiotic resistance of five GLASS pathogens^1^ among populations in conflict and non-conflict affected Middle Eastern countries, described as percentage median resistance and interquartile range (IQR) of data reported in 116 articles

GLASS pathogen	Resistance profile of public health importance	Overall percentage median resistance	Percentage median resistance in conflict-affected countries	Percentage median resistance in non-conflict affected countries	References
***(Total number of isolates)***	***(Total number of isolates tested)***	(IQR)	(IQR)	(IQR)	
***Acinetobacter*** **spp.** ***(6′129)***	Carbapenem-resistant *(5′825)*	74.23 (25.89–88.33)	78.00 (58.97–82.41)	72.73 (23.23–91.30)	[42, 46–48, 61, 63, 65, 68, 80, 83, 85, 88, 90, 92, 97, 99, 101, 104, 106, 108, 110, 115, 123, 124, 126, 131, 133–135, 138, 140, 153, 157, 167, 171, 172]
***E. coli (41′472)***	Carbapenem-resistant *(36′116)*	8.07 (0.54–25.34)	1.33 (0–7.58)	14.29 (5.00–50.00)	[42, 45, 47–49, 54, 59, 65, 67, 71, 75, 76, 87, 89, 91, 99, 105, 107, 109, 32, 6, 114–117, 119, 123, 128, 133, 143, 151, 155, 160, 166, 172]
	ESBL-producing *(35′655)*	32.30 (21.75–50.93)	25.85 (14.99–48.43)	39.21 (27.75–51.94)	[45, 52, 54, 59, 62, 72, 73, 75, 76, 79, 105, 114, 117, 123, 135, 141, 154, 155, 159, 160, 164, 165]
***K. pneumoniae (2′373)***	Carbapenem resistant *(1′341)*	15.39 (2.03–24.67)	17.71 (9.94–26.56)	15.00 (1.21–20.18)	[42, 47, 69, 76, 84, 86, 92, 99, 104, 107, 112, 115–118, 145, 153, 155, 158, 160, 172]
	ESBL-producing *(1′013)*	27.91 (20.39–66.03)	75.46 (70.42–80.49)	22.50 (19.65–38-10)	[52, 62, 72, 76, 117, 155, 158, 160, 164, 165]
***S. aureus (8′886)***	MRSA *(8′403)*	45.08 (25.96–61.57)	43.37 (28.11–59.42)	45.32 (24.44–61.42)	[41, 43, 44, 51–53, 55–57, 65, 66, 71, 74, 77, 78, 81, 95, 96, 102, 111, 116, 121, 123, 125, 128, 129, 133, 135, 136, 139, 144, 148, 150, 161, 163, 168, 170]
	VRSA *(6′170)*	0 (0–10.20)	1.32 (0.08–3.84)	0 (0–16.67)	[44, 51, 65, 70, 71, 89, 95, 96, 103, 116, 123, 128, 133, 134, 139, 142, 144, 150, 168]
***S. pneumoniae (1′766)***	Penicillin non-susceptible *(1′050)*	50.00 (8.78–68.95)	10.90 (5.45–30.45)	55.00 (20.42–77.81)	[50, 64, 93, 99, 120, 127, 152, 156]

^1^ WHO. Global Antimicrobial Resistance Surveillance System (GLASS) Report: Early Implementation 2016–2017. Geneva, Switzerland; 2017

A third (32.3%) of *E. coli* isolates showed ESBL-producing profile, and 8.1% resistance to carbapenems (IQR 0.6–25.3). In both cases, the proportion of resistant isolates appeared to be higher in non-conflict affected countries compared to conflict-affected ones.

Regarding *S. aureus*, the overall median percentage of methicillin-resistance was 45.1% (IQR 26.0–61.6). Unfortunately, the number of articles was too small in conflict affected compared to non-conflict affected countries to make a reliable comparison, and again study populations were very heterogeneous [44, 95, 144]. However, from the few studies included, no major difference was detected between the two contexts.

Proportion of carbapenem resistance among *Acinetobacter spp.* isolates was significant (74.2%) and appeared slightly higher in conflict-affected settings compared to stable countries (78.0% vs 72.7%, respectively).

Only a small proportion of samples of *K. pneumoniae* was tested for carbapenem resistance and ESBL-production (56.5 and 42.7% respectively) and 28.0% of samples appeared to be ESBL producers, with a substantial difference between conflict and non-conflict affected countries (75.5% vs 22.5%, respectively). The carbapenem resistance profile did not vary significantly between the two contexts, with an overall proportion of cases of 15.4%.

As for *S. pneumoniae,* half of all isolates were detected as non-susceptible to penicillin, with substantially higher proportion in stable contexts (55.0%) compared to conflict-affected settings (10.9%).

*Salmonella spp.* and *Shigella spp.* AMR were investigated in too few studies (five and three respectively) to allow a valid summary to be included.

Tables 3 and 4 in the annex offer more details on the number of studies on each pathogen per country in conflict-affected vs non-conflict-affected countries, respectively. Tables 5 and 6 provide details on the number of studies per site of infections in conflict-affected vs non-conflict-affected countries, respectively. From these tables we can observe that studies conducted in non-conflict affected countries appeared to be slightly more focused in terms of site of infection under investigation compared to studies conducted in conflict affected countries, where in a substantial proportion of cases the site of infection was not specified, or the authors were analysing resistance profiles of bacteria isolated from a miscellanea of specimens.

**Table 3:** Number of studies included in this systematic review reporting on individual GLASS pathogens in fragile and conflict affected Countries

GLASS pathogen	Country N* (References)								References
	Djibouti (Tot ***N*** = 0)	Iraq (Tot ***N*** = 24)	Israel/OPT (Tot ***N*** = 7)	Lebanon (Tot ***N*** = 9)	Libya (Tot ***N*** = 6)	Syria (Tot ***N*** = 4)	Yemen (Tot ***N*** = 1)	Total (%) (***N*** = 51)	
*Acinetobacter* spp.	–	4/24	–	6/9	2/6	–	–	**12/51 (23.5%)**	[44, 50, 106, 109, 111, 115, 127, 129, 143, 160, 170, 174]
*E. coli*	–	5/24	2/7	1/9	5/6	3/4	–	**16/51 (31.4%)**	[44, 45, 50, 55, 62, 74, 82, 86, 114, 119, 131, 144, 150, 154, 162, 163]
*K. pneumoniae*	–	6/24	–	–	2/6	–	–	**8/51 (15.7%)**	[89, 91, 109, 117, 122, 161, 163, 165]
*N. gonorrhoeae*	–	–	–	–	–	–	–	**0/51 (0%)**	NA
*Salmonella* spp.	–	1/24	–	2/9	–	–	–	**3/51 (5.9%)**	[50, 140, 152]
*Shigella* spp.	–	1/24	–	1/9	–	–	–	**2/51 (3.9%)**	[50, 66]
*S. aureus*	–	6/24	4/7	2/9	–	2/4	1/1	**15/51 (29.4%)**	[50, 53, 54, 61, 63, 64, 73, 85, 101, 102, 118, 126, 131, 132, 147]
*S. pneumoniae*	–	1/24	1/7	2/9	–	–	1/1	**5/51 (9.8%)**	[50, 58, 126, 130, 159]

* Articles can report antibiotic resistance profile on more than one pathogen, and therefore the sum of the articles in this table can differ from the total per Country detailed

**Table 4:** Number of studies included in this systematic review reporting on individual GLASS pathogens in non-conflict affected Countries

GLASS pathogen	Country N* (References)											References
	Bahrain (Tot *N* = 1)	Egypt (Tot ***N*** = 37)	Jordan (Tot ***N*** = 8)	Kuwait (Tot *N* = 1)	Morocco (Tot *N* = 7)	Oman (Tot *N* = 1)	Qatar (Tot *N* = 0)	KSA (Tot ***N*** = 26)	Tunisia (Tot *N* = 1)	UAE (Tot *N* = 3)	Total (%) (Tot ***N*** = 82)	
*Acinetobacter* spp.	–	11/37	1/8	–	2/7	–	–	13/26	–	–	**27/82 (32.9%)**	[46, 48, 49, 52, 56, 68–70, 72, 75, 87, 90, 92, 95, 97, 98, 103, 105, 113, 134, 136–139, 141, 156, 167]
*E. coli*	–	18/37	3/8	1/1	4/7	–	–	11/26	–	1/3	**38/82 (46.3%)**	[41, 46–49, 52, 57, 60, 69, 71, 72, 78–80, 83, 94, 96, 98, 100, 105, 110, 112, 120, 121, 123, 133, 136, 138, 139, 146, 149, 157, 158, 167–169, 171, 172]
*K. pneumoniae*	–	9/37	–	1/1	4/7	–	–	10/26	–	2/3	**26/82 (31.7%)**	[45, 47, 48, 51, 59, 66, 68, 75, 78, 82, 92, 97, 104, 105, 112, 120, 121, 133, 139, 145, 148, 156, 158, 167, 168, 171]
*N. gonorrhoeae*	–	–	–	–	–	–	–	–	–	–	**0/82 (0%)**	NA
*Salmonella* spp.	–	1/37	–	–	–	–	–	1/26	–	–	**2/82 (0%)**	[48, 104]
*Shigella* spp.	–	1/37	–	–	–	–	–	–	–	–	**1/82 (1.2%)**	[78]
*S. aureus*	–	16/37	3/8	–	1/7	–	–	10/26	–	–	**30/82 (22.7%)**	[51, 59, 60, 65, 71, 72, 77, 78, 81, 84, 88, 96, 107, 108, 116, 120, 125, 128, 136–139, 142, 145, 151, 153, 164, 166, 171, 173]
*S. pneumoniae*	1/1	3/37	1/8	–	1/7	1/1	–	–	1/1	1/3	**7/82**^**¶**^ **(8.5%)**	[71, 99, 105, 124, 130, 135, 155]

* Articles can report antibiotic resistance profile on more than one pathogen, and therefore the sum of the articles in this table can differ from the total per Country detailed

^¶^ Articles can describe results for more than one Country: in such case they are counted only once in the total

**Table 5:** Number of studies on each GLASS pathogen classified by site of infection in fragile and conflict affected Countries

GLASS pathogen	Site of infection (References)									
	Colonization	Urinary Tract Infections	Skin and Soft Tissue Infections	Bloodstream Infections	Respiratory Tract Infections	Gastro-Enteritis	Meningitis	Keratitis	Miscellanea	Not specified
***Acinetobacter*** **spp. (Tot = 12)**	–	–	3 (44, 129, 143)	–	–	–	–	–	5 (106, 109, 111, 127, 160)	4 (50, 115, 170, 174)
***E. coli*** **(Tot = 16)**	–	8 (45, 55, 62, 82, 114, 119, 150, 162)	1 (44)	–	–	2 (74, 86)	–	–	2 (144, 154)	3 (50, 131, 163)
***K. pneumoniae*** **(Tot = 8)**	–	1 (91)	1 (89)	–	1 (165)	–	–	–	3 (109, 117, 122)	2 (161, 163)
***N. gonorrhoeae*** **(Tot = 0)**	–	–	–	–	–	–	–	–	–	–
***Salmonella*** **spp. (Tot = 3)**	–	–	–	1 (140)	–	1 (152)	–	–	–	1 (50)
***Shigella*** **spp. (Tot = 2)**	–	–	–	–	–	1 (66)	–	–	–	1 (50)
***S. aureus*** **(Tot = 15)**	3 (54, 101, 147)	–	1 (102)	–	1 (126)	–	–	–	7 (53, 61, 63, 64, 73, 85, 132)	3 (50, 118, 131)
***S. pneumoniae*** **(Tot = 5)**	1 (58)	–	–	–	1 (126)	–	1 (159)	–	1 (130)	1 (50)

**Table 6:** Number of studies on each GLASS pathogen classified by site of infection in non-conflict affected Countries

GLASS pathogen	Site of infection (References)									
	Colonization	Urinary Tract Infections	Skin and Soft Tissue Infections	Bloodstream Infections	Respiratory Tract Infections	Gastro-Enteritis	Meningitis	Keratitis	Miscellanea	Not specified
***Acinetobacter*** **spp. (Tot = 27)**	2 (90, 98)	1 (167)	2 (52, 68)	5 (48, 49, 72, 136, 156)	2 (69, 105)	–	1 (137)	–	10 (46, 56, 70, 75, 95, 97, 113, 134, 139, 141)	4 (87, 92, 103, 138)
***E. coli*** **(Tot = 38)**	3 (94, 98, 123)	11 (41, 57, 112, 133, 149, 157, 158, 167–169, 172)	1 (52)	6 (48, 49, 72, 78, 79, 136)	4 (69, 71, 96, 105)	–	–	–	10 (46, 47, 60, 80, 83, 100, 120, 121, 139, 171)	3 (110, 138, 146)
***K. pneumoniae*** **(Tot = 26)**	1 (97)	6 (41, 112, 133, 158, 167, 168)	1 (52)	4 (48, 49, 79, 156)	2 (69, 105)	–	–	1 (145)	11 (46, 60, 67, 76, 83, 93, 120, 121, 139, 148, 171)	–
***N. gonorrhoeae*** **(Tot = 0)**	–	–	–	–	–	–	–	–	–	–
***Salmonella*** **spp. (Tot = 2)**	–	–	–	1 (48)	–	1 (104)	–	–	–	–
***Shigella*** **spp. (Tot = 1)**	–	–	–	1 (78)	–	–	–	–	–	–
***S. aureus*** **(Tot = 30)**	11 (51, 59, 65, 81, 84, 88, 107, 116, 151, 166, 173)	–	3 (125, 128, 164)	5 (72, 78, 108, 136, 153)	2 (71, 96)	–	1 (137)	1 (145)	6 (60, 77, 120, 139, 142, 171)	1 (138)
***S. pneumoniae*** **(Tot = 7)**	2 (99, 135)	–	–	–	2 (71, 105)	–	–	–	3 (124, 130, 155)	–

**Fig. 3:**
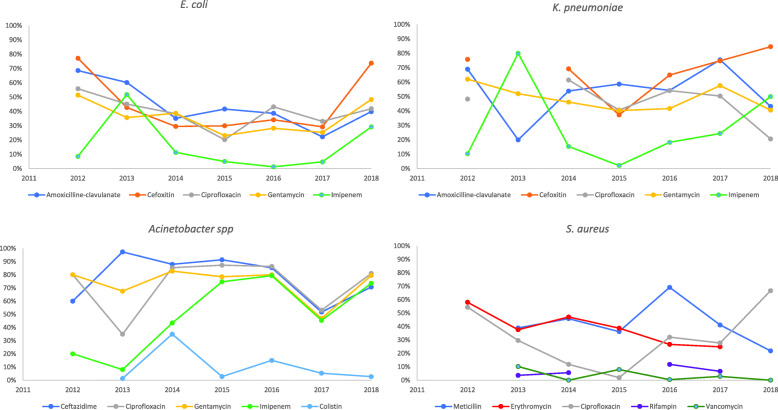
Temporal trends in the proportion of resistance to five classes of antibiotics by year of publication among studies including *E. coli*, *K. pneumoniae*, *Acinetobacter spp*. and *S. aureus*

Figure 3 presents the temporal trends in antibiotic resistances to the most commonly used classes of antibiotics for the four most frequently reported pathogens (*E. coli*, *K. pneumoniae*, *Acinetobacter spp*. and *S. aureus*), tracked by year of publication.

**Fig. 4:**
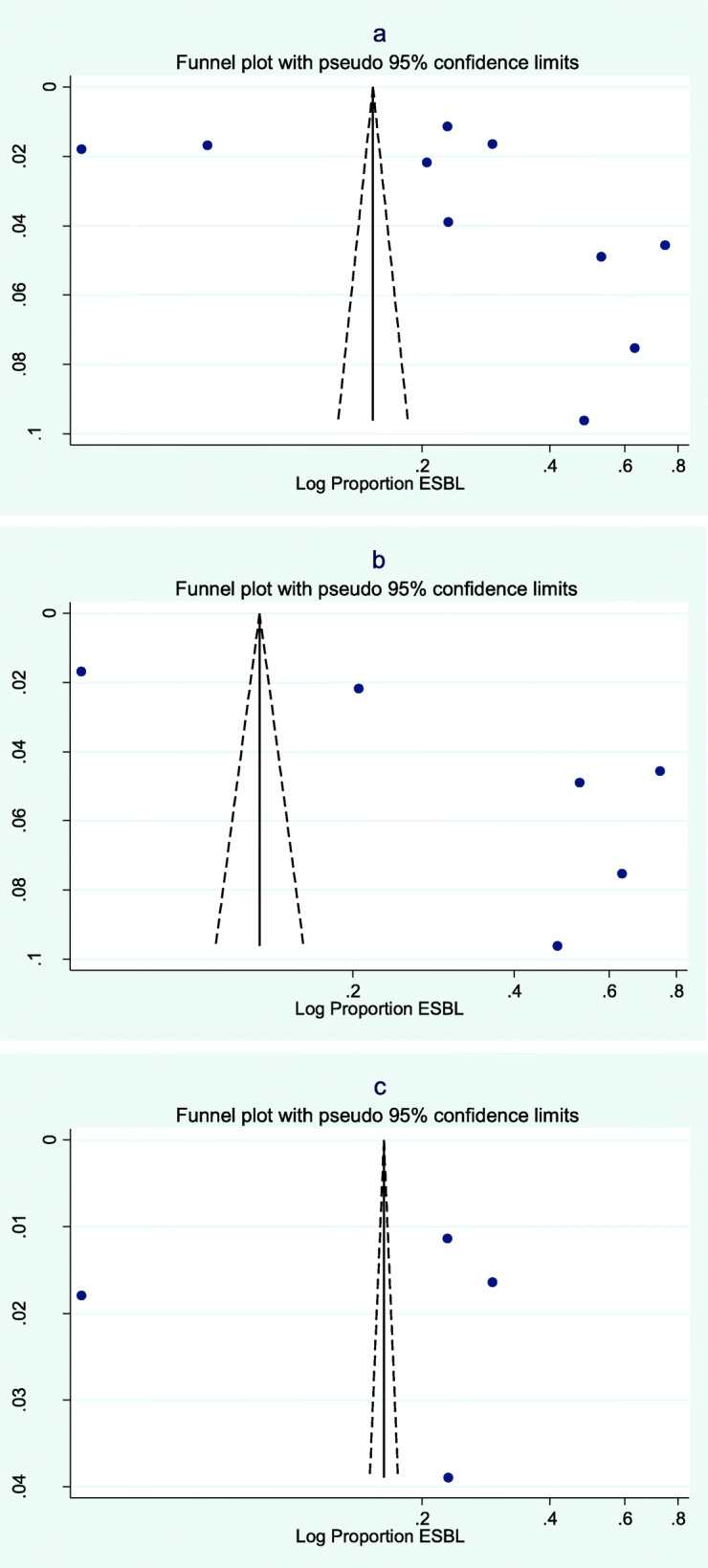
Funnel plots for publication bias in ten studies reporting the proportion of ESBL producers among *E. coli* isolated from urinary samples: a) All studies (Eggers regression test *p* = 0.52), b) Studies in conflict settings (Eggers regression test *p* = 0.78), c) Studies in non-conflict settings (Eggers regression test *p* = 0.07)

Publication bias was assessed for a sub-sample of ten studies which presented the proportion of ESBL producers among *E. coli* isolates on urinary samples, six of which were conducted in conflict-affected countries [45, 54, 59, 75, 114, 159] and four in non-conflict settings [154, 155, 164, 165]. Figure 4 shows the funnel plots elaborated on the logarithm of the proportion of ESBL producers amongst *E. coli* isolates in urine samples in all studies, and in studies conducted in conflict and non-conflict settings separately. The funnel plots show asymmetry and therefore would support the hypothesis of publication bias; however, Egger’s regression test showed absence of statistical evidence of publication bias overall (*p*-value 0.52), as well as specifically for studies conducted in non-conflict and conflict settings (*p*-values 0.78 and 0.07 respectively).

## Discussion

While it is undoubted that AMR constitutes one of the most dangerous threats to public health on a global scale, the scientific community has reached no consensus yet on how to estimate and quantify the burden of AMR [175, 176]. This can be partly explained by the complex interactions between upstream and downstream determinants of AMR, which require a system level analysis through conceptual frameworks that need to be both context-specific and coherent at global level [177, 178].

In terms of upstream determinants, the Middle Eastern conflict-affected countries are low and middle income countries, while stable settings, particularly the Gulf Cooperation Council States, are high income economies [179]. These differences will influence the affordability to enforce measures, laws, and regulations regarding AMR. Beside the differences in wealth macro-indicators, common traits among the different countries emerge from the literature when investigating more proximal determinants of AMR, such as antibiotic prescription and self-medication practices [180, 181].

The interplay between the different determinants can be more pronouncedly hard to understand in fragile settings, particularly in situations of armed conflict, where additional drivers of AMR can complicate the picture. This has been insightfully analysed by Abbara and colleagues in a recent series of papers, in which it is described how the Syrian conflict has exacerbated the risk of AMR emergence and spread among the affected populations at a local level, and on a more global scene when considering the number of countries in which those fleeing are seeking refuge [182, 183].

### Summary of evidence

Only six out of the 18 countries included in this systematic review have adhered to the GLASS (Bahrain, Egypt, Lebanon, Oman, KSA, and UAE) and are at different stages of the development or implementation of their National Action Plan on AMR, and amongst these only one – Lebanon – is considered a fragile context [184]. The substantial number of articles retrieved in this review denotes an active research on the topic in the region, both in conflict and non-conflict affected settings. However, the vast majority of evidence comes from only three countries: Egypt, KSA, and Iraq.

The 132 studies included in this review present heterogeneity and inconsistencies in terms of study designs, laboratory methodologies and standards adopted, and a variety of clinical scenarios included. The lack of harmonization in the regional surveillance system is not a prerogative of the Middle East, as it has been highlighted also in reviews conducted in other geographical areas outside the Western countries [10, 11, 13].

The majority of the included articles were cross-sectional studies, focusing on proportion and prevalence as outcome measures, with only two studies reporting incidence measures [131, 161]. Moreover, the sources of data being limited to hospitals and hospital laboratories, do not allow to describe the burden of AMR at community and primary care level, where more than 80% of the total volume of antibiotics is used according to Western estimates [185], and where antibiotic prescription and consumption practices are less regulated than in hospital settings [186], particularly in the Middle East [187, 188].

The most frequently reported GLASS pathogens in the studies included in this review were those belonging to the *Enterobacteriaceae* family (*E. coli* and *K. pneumoniae*), *Acinetobacter spp.*, and *S. aureus,* both in conflict and non-conflict affected countries (see Tables 3 and 4).

Where possible, the resistance profiles identified were compared with those of the same micro-organisms reported in the European Antimicrobial Resistance Surveillance Network (EARS-Net) [189], particularly those from European Mediterranean countries, as this is geographically the closest example of consolidated and homogeneous data collection system on ABR to the Middle East.

The proportion of ABR emerging from the studies included in this review is concerning. In fact, among the *Enterobacteriaceae* included, around one third of the isolates showed an ESBL-producing profile. *K. pneumoniae* in conflict-affected countries appeared to have higher degrees of resistance to third generation cephalosporin, however random variation cannot be ruled out considering the limited number of studies conducted in these settings. The highest proportion of studies on both *E. coli* and *K. pneumoniae* were related to urinary tract infections, both in conflict and non-conflict affected countries (Tables 5 and 6). The carbapenem-resistance proportion among GLASS *Enterobacteriaceae* included in this review appear to be much higher than what is described in European settings and in the Mediterranean region [189, 190], however it is important to reiterate that the vast majority of studies included were hospital based, and investigating AMR in patients with underlying medical conditions, who are notoriously at higher risk of harbouring MDR strains.

The methicillin-resistance patterns in *S. aureus* detected in this review are similar to those described in a specific review on MRSA prevalence in the Middle East and North Africa [191], and the proportion of MRSA over the total number of *S. aureus* isolates appears to be higher than what described in other geographical regions such as Africa [192] and Europe, although again close to the figures of the European Mediterranean countries [189]. Among the articles reporting the AMR profile of *S. aureus,* nasal carriage was the most frequently studied condition (Tables 5 and 6).

The figures of carbapenem-resistance among *Acinetobacter spp.* are comparable with those of Southern Europe, particularly Spain, Italy and Greece, where the proportion vary between 69.9 and 94.9% [189]. While three out of the 12 studies on *Acinetobacter spp.* in conflict affected countries were associated with skin and soft tissue infections, only two out of the 27 retrieved from non-conflict affected settings were investigating this specific site of infection (see Tables 5 and 6). MDR *Acinetobacter baumannii* in particular has been described in the Middle East as associated with war-related wounds [26] already since the USA military interventions in Afghanistan and Iraq [193]. This has re-emerged recently in the literature related to wounded Syrian civilians since the onset of the Syrian crisis [194, 195], and confirmed also in the study by Rafei et al. included in this review [101], in which Syrian refugees admitted mostly for war-related wounds to a Lebanese hospital were significantly more likely (*p* < 0.001) to harbour carbapenem-resistant strains than Lebanese patients (74% versus 47%). This peculiar feature, particularly in conflict-affected settings, deserves undoubtedly further investigation.

*S. pneumoniae* was less represented than other pathogens in this review, however a sufficient number of studies allowed to attempt a quantitative summary of the proportion of penicillin resistance observed: the findings of this review are consistent with those of other studies conducted in the region, and confirm that penicillin non-susceptibility among *S. pneumoniae* in the Middle East is frequently reported around 50% [196].

No specific temporal trends were identified in the reports of antibiotic resistances to the most commonly used antibiotics among the four most frequently reported GLASS pathogens included in this systematic review (Fig. 3). However, caution must be applied when interpreting these results as no conclusive inference can be made on these data. In fact, the temporal trends were tracked exclusively by year of publication, and therefore might be relative to different years of sample collection: such detail was not consistently specified in the included papers, and where it was, it collated data on samples collected over several years, with the authors having no access to the raw data. Moreover, the studies included were heterogeneous in terms of sampling strategy, sample collection sites, laboratory methodology, and interpretation criteria adopted.

It is worth to underline the lack of research about *N. gonorrhoeae* in this region: this might be due to cultural and social aspects surrounding sexual and reproductive health knowledge, attitude, practices and taboos related to healthcare seeking for sexually transmitted infections (STIs) in the Middle East. The knowledge gap on STIs in this region is still significant, despite the recently describe increase in focus [197], and more research is needed to understand the prevalence of STIs and AMR related to them.

It is remarkable that a substantial proportion of studies were not investigating specific clinical conditions but were rather reporting unspecified or miscellaneous sites of infection (Tables 5 and 6): this is probably linked to the convenience sampling almost exclusively used in the research design of the included articles, and therefore to the poor methodological rigour of the evidence available from this geographical context.

### Specific considerations on conflict-affected settings

With the currently available evidence it is not possible to draw definitive conclusions on whether the burden of AMR is differential between conflict and non-conflict affected settings in the Middle East, as different pathogens show conflicting results. The inconclusive findings of this review mirror those of similar studies conducted on AMR in *M. tuberculosis* among conflict-affected populations, which similarly failed to detect a significant difference in the levels of resistances to antimicrobials when comparing displaced populations to populations in the home countries [198].

Several elements can potentially explain the lack of differences detected between stable countries and conflict-affected countries in this review. First of all, studies from conflict-affected settings tend to be less precise in terms of completeness of information provided, and more focused on adult populations without severe comorbidities. Secondly, in conflict-affected settings the availability and accessibility of antibiotics could be severely compromised, thus minimizing the selective pressure on bacteria for the development of resistances. Thirdly, publication bias, although not statistically evident in this systematic review, might limit the availability of evidence from fragile contexts, due either to lack of access of researchers to the most at risk populations or to prioritization of research on most pressing public health needs and/or life-threatening conditions (such as war trauma or acute malnutrition). The visual evidence of publication bias that emerges from the funnel plots is contradicted by the results of the Egger’s test. Caution must be applied when interpreting these results, as there are well described limitations with the use of funnel plots to assess publication bias in this type of systematic review [199]. Lastly, there might be less awareness, both among health authorities and humanitarian health organizations delivering health services in conflict-affected countries, of the potential public health threat that an unregulated and uncontrolled distribution of antibiotics might represent for the population.

In summary, the pathways through which conflict and non-conflict-affected countries can develop antibiotic resistances might differ based on the social, economic and political context. This stresses the importance of strengthening national and regional surveillance systems and adopting harmonized approaches that tackle antibiotic resistances at all levels, from agriculture and food safety to veterinary medicine, from legislative regulation to improved quality of scientific research, through a One Health approach as advocated by WHO.

### Limitations

This systematic review presents some limitations that need to be taken into account when interpreting the results described.

From a methodological perspective, only one author screened and selected the available literature applying inclusion and exclusion criteria: while this is not aligned with the best practices required to present a systematic review, it was due to the nature and the specific requirements of a project that started as an individual work within an academic path, and it was mitigated by an independent supervision by the second author in view of the peer review process.

From the perspective of completeness of data and qualitative analysis of the results, there was no data collection on clinical outcomes - such as mortality - which was a choice dictated by the fact that the available literature was mainly laboratory based and vastly lacking in this type of detail.

Another critical element missing from this review is the proportion of multi drug resistant microorganisms among those identified: this was not included by virtue of the lack of reporting and/or the lack of access to raw data from the included studies where reported.

The strict focus on GLASS bacteria might have led to overlook important pathogens, bacterial and non-bacterial, that might be relevant to the Middle Eastern context, such as *Pseudomonas aeruginosa* [200–202]*, Neisseria meningitidis* [203]*,* or viral infections – such as respiratory viruses – that are prevalent and of significant public health importance in this context [204].

From a standpoint of completeness and validity of the evidence retrieved, there are several elements that need to be mentioned as caveats before making any inference on the magnitude of the problem of AMR resistances in the Middle East. One element that needs to be mentioned is the fact that grey literature was not specifically searched for this review, and therefore some evidence might not be represented in the presented results. This was primarily due to the difficulties in retrieving unpublished reports particularly in conflict-affected settings. The main aspect to be cautiously appraised lies in the methodological weaknesses of the studies included in this review, including sampling. It is not possible in fact to rule out sampling variation as first possible explanation of the magnitude of proportions of AMR observed. The hospital-based cross-sectional study design dominating the published literature on the topic adds uncertainty to the validity and the generalizability of findings to the wider Middle Eastern population.

The limitations generated by these factors might be further impaired by the high risk of selection bias, information bias, misclassification, and the limited control for the potential confounding effect of socio-demographic variables detected in a considerable proportion of studies (Additional file 1: Appendix 4).

The strict inclusion and exclusion criteria applied, along with the descriptive analysis performed, that takes into account the variability observed and is methodologically aligned with that reported in similar studies in other contexts [11], are an attempt to both provide a reliable summary of the evidence available and make the findings interpretable and comparable from the perspective of scarce homogeneity and systematization of knowledge on the issue of AMR in resource limited settings.

The consistency of the findings described with those available in the literature on specific pathogens in the region leads to hypothesize that, despite the limitations above described, the concerning high proportion of AMR strains detected in this review has a certain degree of validity.

## Conclusions

Based on the findings described in this systematic review, the first aspect that emerges is the clear lack of standardization in the methodological approach to AMR research in the Middle East, which hinders any possibility of drawing conclusions on the incidence or prevalence of specific resistance patterns at population level in the Middle East. This is mainly due to the predominantly hospital-based, cross-sectional nature of the studies performed, and is further aggravated by some methodological aspects: first of all, the convenience sampling almost universally adopted to investigate AMR, and secondly the lack of stratification or other statistical approaches to control for the multiple potential confounding effects that can play a role in the emergence and spread of AMR.

From what emerges from the descriptive analysis offered as a summary in this review, the proportion of antibiotic resistances among specific GLASS pathogens is concerning, particularly in the case of *Acinetobacter spp.*, which deserves further investigation, particularly considering its predominance in war-related wounds.

However, it is not possible to draw any conclusion on the hypothesized differences in the magnitude of the problem of AMR between conflict and non-conflict affected countries, due to the flaws above described.

It is recommended that Middle Eastern country who have not done so yet, join the second phase of implementation of GLASS: to facilitate this, an Arabic version of both the GLASS website and GLASS manual will be needed.

The Middle Eastern scientific community should also adhere to the standardized methods promoted and supported by GLASS for AMR research and reporting, in order to improve the accuracy, quality and comparability of data collected on AMR in this region. Such harmonization effort will allow a more in-depth understanding of the drivers and patterns of acquisition and transmission of antibiotic resistances in the Middle East.

A stronger focus on population-based and primary care-based research will be needed, in order to avoid capturing exclusively high-risk populations in hospital settings, which could distort the findings. Such knowledge is necessary for improving national and local treatment guidelines, tailoring them to the specificities of each context.

Governments, academics, as well as aid agencies in conflict-affected countries, should try in parallel to promote research on and implementation of appropriate antibiotic resistance surveillance system and antibiotic stewardship programs at all levels of care, in order to timely tackle the threat of AMR through optimization of antibiotic prescription and consumption practices based on the resistance profiles circulating in this region.

## Supplementary Information

**Additional file 1:**
**Appendix 1.** PRISMA checklist.
**Appendix 2.** Search strategy.
**Appendix 3.** Data extraction form.
**Appendix 4.** Summary of the assessment of risk of bias.

## Data Availability

The data that support the findings of this study are available upon request to the corresponding author.

## Abbreviations

ABR: Antibiotic resistance
AIDS: Acquired Immune Deficiency Syndrome
AMR: Antimicrobial resistance
API: Analytical Profile Index
AST: Antibiotic Susceptibility Testing
BSAC: British Society for Antimicrobial Chemotherapy
CA-SFM: *Comité de l’Antibiogramme de la Societé Française de Microbiologie*
CDC: Centre for Disease Prevention and Control
CLSI: Clinical and Laboratory Standards Institute
EARS-net: European Antibiotic Resistance Surveillance Network
ECDC: European Centre for Disease Control
EMR: Eastern Mediterranean Region
ESBL: Extended Spectrum Beta Lactamase
EUCAST: European Committee on Antimicrobial Susceptibility Testing
GLASS: Global Antimicrobial Resistance Surveillance System
HIV: Human Immunodeficiency Virus
IMEMR: Index Medicus for the Eastern Mediterranean Region
IQR: Interquartile Range
KSA: Kingdom of Saudi Arabia
MDR: Multidrug Resistant
MIC: Minimum Inhibitory Concentration
MRSA: Methicillin-Resistant *Staphylococcus aureus*
MSF: *Médecins sans Frontières*
NCDs: Non-Communicable Diseases
PCR: Polymerase Chain Reaction
PRISMA: Preferred Reporting Items for Systematic Reviews and Meta-Analysis
STIs: Sexually Transmitted Infections
UAE: United Arab Emirates
VRSA: Vancomycin-Resistant *Staphylococcus aureus*
WHA: World Health Assembly
WHO: World Health Organization
