# Supplementary material for: Antimicrobial resistance among GLASS pathogens in conflict and non-conflict affected settings in the Middle East: a systematic review
Source: BMC Infect Dis. 2020 Dec 9;20:936. doi: 10.1186/s12879-020-05503-8 (PMC7724697; doi:10.1186/s12879-020-05503-8)
Supplement: Supplementary file 1 — Additional file 1: Appendix 1. PRISMA checklist. Appendix 2. Search strategy. Appendix 3. Data extraction form. Appendix 4. Summary of the assessment of risk of bias. [file 12879_2020_5503_MOESM1_ESM.docx]

# Appendices

## Appendix 1. PRISMA checklist

| **Section/topic** | **#** | **Checklist item** | **Page #** |
| --- | --- | --- | --- |
| **TITLE** | | | |
| Title | 1 | Identify the report as a systematic review, meta-analysis, or both. | 1 |
| **ABSTRACT** | | | |
| Structured summary | 2 | Provide a structured summary including, as applicable: background; objectives; data sources; study eligibility criteria, participants, and interventions; study appraisal and synthesis methods; results; limitations; conclusions and implications of key findings; systematic review registration number. | 1-2 |
| **INTRODUCTION** | | |  |
| Rationale | 3 | Describe the rationale for the review in the context of what is already known. | 1-3 |
| Objectives | 4 | Provide an explicit statement of questions being addressed with reference to participants, interventions, comparisons, outcomes, and study design (PICOS). | 4 |
| **METHODS** | | | |
| Protocol and registration | 5 | Indicate if a review protocol exists, if and where it can be accessed (e.g., Web address), and, if available, provide registration information including registration number. | N/A |
| Eligibility criteria | 6 | Specify study characteristics (e.g., PICOS, length of follow-up) and report characteristics (e.g., years considered, language, publication status) used as criteria for eligibility, giving rationale. | 6 |
| Information sources | 7 | Describe all information sources (e.g., databases with dates of coverage, contact with study authors to identify additional studies) in the search and date last searched. | 4-5 |
| Search | 8 | Present full electronic search strategy for at least one database, including any limits used, such that it could be repeated. | Appendix 2 |
| Study selection | 9 | State the process for selecting studies (i.e., screening, eligibility, included in systematic review, and, if applicable, included in the meta-analysis). | 5 |
| Data collection process | 10 | Describe method of data extraction from reports (e.g., piloted forms, independently, in duplicate) and any processes for obtaining and confirming data from investigators. | 7-8 |
| Data items | 11 | List and define all variables for which data were sought (e.g., PICOS, funding sources) and any assumptions and simplifications made. | Appendix 3 |
| Risk of bias in individual studies | 12 | Describe methods used for assessing risk of bias of individual studies (including specification of whether this was done at the study or outcome level), and how this information is to be used in any data synthesis. | 8-9 |
| Summary measures | 13 | State the principal summary measures (e.g., risk ratio, difference in means). | 7 |
| Synthesis of results | 14 | Describe the methods of handling data and combining results of studies, if done, including measures of consistency (e.g., I^2^) for each meta-analysis. | 8 |
| Risk of bias across studies | 15 | Specify any assessment of risk of bias that may affect the cumulative evidence (e.g., publication bias, selective reporting within studies). | 8 |
| Additional analyses | 16 | Describe methods of additional analyses (e.g., sensitivity or subgroup analyses, meta-regression), if done, indicating which were pre-specified. | N/A |
| **RESULTS** | | | |
| Study selection | 17 | Give numbers of studies screened, assessed for eligibility, and included in the review, with reasons for exclusions at each stage, ideally with a flow diagram. | 9-12; Figure 2 |
| Study characteristics | 18 | For each study, present characteristics for which data were extracted (e.g., study size, PICOS, follow-up period) and provide the citations. | 13 |
| Risk of bias within studies | 19 | Present data on risk of bias of each study and, if available, any outcome level assessment (see item 12). | 14-15 |
| Results of individual studies | 20 | For all outcomes considered (benefits or harms), present, for each study: (a) simple summary data for each intervention group (b) effect estimates and confidence intervals, ideally with a forest plot. | 16-19 |
| Synthesis of results | 21 | Present the main results of the review. If meta-analyses are done, include for each, confidence intervals and measures of consistency. | 16-19 |
| Risk of bias across studies | 22 | Present results of any assessment of risk of bias across studies (see Item 15). | N/A |
| Additional analysis | 23 | Give results of additional analyses, if done (e.g., sensitivity or subgroup analyses, meta-regression [see Item 16]). | N/A |
| **DISCUSSION** | | | |
| Summary of evidence | 24 | Summarize the main findings including the strength of evidence for each main outcome; consider their relevance to key groups (e.g., healthcare providers, users, and policy makers). | 20-24 |
| Limitations | 25 | Discuss limitations at study and outcome level (e.g., risk of bias), and at review-level (e.g., incomplete retrieval of identified research, reporting bias). | 25-26 |
| Conclusions | 26 | Provide a general interpretation of the results in the context of other evidence, and implications for future research. | 26-28 |
| **FUNDING** | | | |
| Funding | 27 | Describe sources of funding for the systematic review and other support (e.g., supply of data); role of funders for the systematic review. | 31 |

## Appendix 2. Search strategy

**Medline, extended to EMBASE and Global Health via Ovid**

| **Item** | **Search terms** |
| --- | --- |
| **#1** | exp drug resistance, microbial/ or exp drug resistance, bacterial/ |
| **#2** | (bacterial resistan* OR antibiotic-resistan* OR antimicrobial* resistan* OR anti-microbial* resistan* OR microbial-resistan* OR multi-drug* resistan* OR multidrug-resistan* OR bacterial susceptib* OR antibiotic* susceptib* OR antimicrobial* susceptib* OR anti-microbial* susceptib* OR microbial* susceptib* OR multi-drug* susceptib* OR multidrug* susceptib* OR microbial* decrease* susceptib* OR  antibiotic* decrease* susceptib* OR antimicrobial* decrease* susceptib* OR  anti-microbial* decrease* susceptib* OR beta-lactamase* OR ESBL OR metallo-beta-lactamase* OR carbapenem-resistan* OR methicillin-resistan* OR MRSA OR vancomycin-resistan* OR fluoroquinolone-resistan* OR cephalosporin-resistan*).mp. |
| **#3** | 1 OR 2 |
| **#4** | exp africa, northern/ or exp middle east/ |
| **#5** | (Middle East* OR North* Africa OR East* Mediterranean OR Bahrain OR Djibouti OR Egypt* OR Iraq* OR Israel* OR Palestin* OR Occupied Palestin* Territor* OR Gaza strip OR West Bank OR Jordan* OR Kuwait OR Leban* OR Libya* OR Morocc* OR Oman OR Qatar* OR Saudi Arabia OR Syria* OR Tunisia* OR United Arab Emirates OR Yemen*).mp. |
| **#6** | 4 OR 5 |
| **#7** | 3 AND 6 |
| **#8** | limit 7 to (yr="2011 -Current" AND (arabic OR english OR french OR italian OR portuguese OR spanish)) |

**PubMed**

| **Item** | **Search terms** |
| --- | --- |
| **#1** | "Drug Resistance, Microbial"[Mesh]) OR "Drug Resistance, Bacterial"[Mesh] |
| **#2** | (bacterial resistan*[Title/Abstract] OR antibiotic-resistan*[Title/Abstract] OR antimicrobial* resistan*[Title/Abstract] OR anti-microbial* resistan*[Title/Abstract] OR microbial-resistan*[Title/Abstract] OR multi-drug* resistan*[Title/Abstract] OR multidrug-resistan*[Title/Abstract] OR bacterial susceptib*[Title/Abstract] OR antibiotic* susceptib*[Title/Abstract] OR antimicrobial* susceptib*[Title/Abstract] OR anti-microbial* susceptib*[Title/Abstract] OR microbial* susceptib*[Title/Abstract] OR multi-drug* susceptib*[Title/Abstract] OR multidrug* susceptib*[Title/Abstract] OR microbial* decrease* susceptib*[Title/Abstract] OR antibiotic* decrease* susceptib*[Title/Abstract] OR antimicrobial* decrease* susceptib*[Title/Abstract] OR anti-microbial* decrease* susceptib*[Title/Abstract] OR beta-lactamase*[Title/Abstract] OR ESBL[Title/Abstract] OR metallo-beta-lactamase*[Title/Abstract] OR carbapenem-resistan*[Title/Abstract] OR methicillin-resistan*[Title/Abstract] OR MRSA[Title/Abstract] OR vancomycin-resistan*[Title/Abstract] OR fluoroquinolone-resistan*[Title/Abstract] OR cephalosporin-resistan*[Title/Abstract]) |
| **#3** | 1 OR 2 |
| **#4** | ("Middle East"[Mesh]) OR "Africa, Northern"[Mesh] |
| **#5** | (Middle East*[Title/Abstract] OR North* Africa[Title/Abstract] OR East* Mediterranean[Title/Abstract] OR Bahrain[Title/Abstract] OR Djibouti[Title/Abstract] OR Egypt*[Title/Abstract] OR Iraq*[Title/Abstract] OR Israel*[Title/Abstract] OR Palestin*[Title/Abstract] OR Occupied Palestin* Territor*[Title/Abstract] OR Gaza strip[Title/Abstract] OR West Bank[Title/Abstract] OR Jordan*[Title/Abstract] OR Kuwait[Title/Abstract] OR Leban*[Title/Abstract] OR Libya*[Title/Abstract] OR Morocc*[Title/Abstract] OR Oman[Title/Abstract] OR Qatar*[Title/Abstract] OR Saudi Arabia[Title/Abstract] OR Syria*[Title/Abstract] OR Tunisia*[Title/Abstract] OR United Arab Emirates[Title/Abstract] OR Yemen*[Title/Abstract]) |
| **#6** | 4 OR 5 |
| **#7** | 3 AND 6 |
| **#8** | 7 AND Publication date from 2011/01/01 to 2018/06/21 ; Languages: Arabic, English, French, Italian, Portuguese, Spanish |

## Appendix 3. Data extraction form

| **Article information** | - First author - Year of publication - Year(s) of data collection - Country in which the study was conducted (+ conflict-affected vs non-conflict affected at the time data were collected) |
| --- | --- |
| **Study design and methods** | - Type of study - Study setting [hospital, primary healthcare, community] - Population studied [+ conflict-affected vs non-conflict affected at the time data were collected] - Sample size - Age group [children <18 years; adults =>18 years] - Number of samples collected - Clinical syndrome(s) - Primary outcome measure |
| **Antimicrobial resistances** | - Pathogen(s) isolated - Laboratory methodology for pathogen identification - Laboratory methodology for determination of drug susceptibility - Criteria used for interpretation of resistances - AMR profile [antibiotic(s) tested; prevalence of AMR microorganisms or proportion of R samples] |
| **Additional comments** |  |

**Overall quality appraisal for all studies**

| **Items** | **Rating** | **Description** |
| --- | --- | --- |
| The type of study is clearly stated by the author(s) |  |  |
| The type of study is clear from what is written in the article |  |  |
| The research question is clearly stated |  |  |
| The study objectives are clearly described |  |  |
| The outcome measures are clearly described |  |  |
| The characteristics of the study population are clearly described |  |  |
| The study is clearly described in terms of setting, location and period of observation |  |  |
| Sample size and sampling strategy are clearly described |  |  |
| The study provides estimates of random variability in the data for the main outcome |  |  |

**Risk of bias for cohort studies**

| **Items** | **Rating** | **Description** |
| --- | --- | --- |
| **Selection** |  |  |
| - Cohort truly representative of source population(s) - Ascertainment of exposure through secure records - Demonstration that outcome of interest was not present at start of study |  |  |
| **Comparability** |  |  |
| - Statistical methods described and appropriate - Adequate control for confounding |  |  |
| **Outcome** |  |  |
| - Assessment of outcome independent and blind - Follow up long enough for outcomes to occur - Follow up of cohorts complete |  |  |

**Risk of bias for cross sectional studies**

| **Items** | **Rating** | **Description** |
| --- | --- | --- |
| **Selection** |  |  |
| - Sample representative of the population from which it was recruited - Exposure status measured in standard, valid and reliable way - Outcome status measured in standard, valid and reliable way |  |  |
| **Comparability** |  |  |
| - Statistical methods described and appropriate - Adequate control for confounding |  |  |
| **Outcome** |  |  |
| - Missing data taken into account - Sources of bias mentioned |  |  |

**Risk of bias for case-control studies**

| **Items** | **Rating** | **Description** |
| --- | --- | --- |
| **Selection** |  |  |
| - Adequate case definition - Representativeness of the cases - Adequate selection of controls |  |  |
| **Comparability** |  |  |
| - Statistical methods described and appropriate - Adequate control for confounding |  |  |
| **Exposure** |  |  |
| - Ascertainment of exposure valid and reliable - Same method of ascertainment for cases and controls - Non-response rate |  |  |

## Appendix 4. Summary of the assessment of risk of bias

| **Article**  **(Author, Year)** | **Assessment of risk of bias per category** | | | | | | | | | | | | | **Overall risk of bias** |
| --- | --- | --- | --- | --- | --- | --- | --- | --- | --- | --- | --- | --- | --- | --- |
| **Cohort studies** | **Selection** | | | | | | **Comparability** | | | **Outcome** | | | |  |
|  | Cohort representative of source population | Ascertainment of exposure through secure records | | Outcome not present at start of the study | | | Adequate control for confounding | Statistical mehtods described and appropriate | | Indipendent / blind assessment of outcome | Follow up length | | Follow up completeness |  |
| See et al. 2013 | ● | ● | | ● | | | ● | ● | | ● | ● | | ● | Low |
| Sharaf et al. 2016 | ● | ● | | ● | | | ● | ● | | ● | ● | | ● | High |
| Älgå et al. 2018 | ● | ● | | ● | | | ● | ● | | ● | ● | | ● | Low |
| **Case-control studies** | **Selection** | | | | | | **Comparability** | | | **Outcome** | | | |  |
|  | Case definition | Representativeness of cases | | Selection of controls | | | Adequate control for confounding | Statistical mehtods described and appropriate | | Ascertainment of exposure valid and reliable | Same ascertainment for cases and control | | Non response |  |
| Alzoubi et al. 2014 | ● | ● | | ● | | | ● | ● | | ● | ● | | ● | High |
| Dash et al. 2018 | ● | ● | | ● | | | ● | ● | | ● | ● | | ● | Moderate |
| **Cross-sectional studies** | **Selection** | | | | | | **Comparability** | | | **Outcome** | | | |  |
|  | Sample representative of target population | | Exposure measurement valid and reliable | | Outcome measurement valid and reliable | Adequate control for confounding | | | Statistical methods described and appropriate | Missing data taken into account | | Sources of bias explicitely mentioned | |  |
| Abdalla et al. 2012 | ● | | ● | | ● | ● | | | ● | ● | | ● | | High |
| Esmat et al. 2012 | ● | | ● | | ● | ● | | | ● | ● | | ● | | High |
| Adwan G. et al. 2013 | ● | | ● | | ● | ● | | | ● | ● | | ● | | High |
| Adwan K. et al. 2013 | ● | | ● | | ● | ● | | | ● | ● | | ● | | High |
| Al Assil et al. 2013 | ● | | ● | | ● | ● | | | ● | ● | | ● | | Moderate |
| Asaad et al. 2013 | ● | | ● | | ● | ● | | | ● | ● | | ● | | High |
| Aziz et al. 2013 | ● | | ● | | ● | ● | | | ● | ● | | ● | | High |
| Dau et al. 2013 | ● | | ● | | ● | ● | | | ● | ● | | ● | | High |

Summary of the assessment of risk of bias *(cont.)*

| **Cross-sectional studies** | **Selection** | | | | | **Comparability** | | **Outcome** | |  |
| --- | --- | --- | --- | --- | --- | --- | --- | --- | --- | --- |
|  | Sample representative of target population | | Exposure measurement valid and reliable | Outcome measurement valid and reliable | Adequate control for confounding | | Statistical methods described and appropriate | Missing data taken into account | Sources of bias explicitely mentioned |  |
| Faris 2013 | | ● | ● | ● | ● | | ● | ● | ● | High |
| Nasereddin et al. 2013 | | ● | ● | ● | ● | | ● | ● | ● | High |
| Saadabi et al. 2013 | | ● | ● | ● | ● | | ● | ● | ● | High |
| Taha 2013 | | ● | ● | ● | ● | | ● | ● | ● | High |
| Adwan K et al. 2014 | | ● | ● | ● | ● | | ● | ● | ● | High |
| Al-Akydy et al. 2014 | | ● | ● | ● | ● | | ● | ● | ● | High |
| Al-Zaidi et al. 2014 | | ● | ● | ● | ● | | ● | ● | ● | High |
| Auda 2014 | | ● | ● | ● | ● | | ● | ● | ● | High |
| Buzayan et al. 2014 | | ● | ● | ● | ● | | ● | ● | ● | High |
| Dash et al. 2014 | | ● | ● | ● | ● | | ● | ● | ● | High |
| El-Din et al. 2014 | | ● | ● | ● | ● | | ● | ● | ● | High |
| Elfaky et al. 2014 | | ● | ● | ● | ● | | ● | ● | ● | High |
| Es-Said, Elfazi et al. 2014 | | ● | ● | ● | ● | | ● | ● | ● | High |
| Es-Said, Mahdoufi et al. 2014 | | ● | ● | ● | ● | | ● | ● | ● | High |
| Fattouh et al. 2014 | | ● | ● | ● | ● | | ● | ● | ● | Moderate |
| Harastani et al. 2014 | | ● | ● | ● | ● | | ● | ● | ● | High |
| Ibrahim et al. 2014 | | ● | ● | ● | ● | | ● | ● | ● | High |
| Nageeb et al. 2014 | | ● | ● | ● | ● | | ● | ● | ● | High |
| Rahim et al. 2014 | | ● | ● | ● | ● | | ● | ● | ● | High |
| Salem Bekhit 2014 | | ● | ● | ● | ● | | ● | ● | ● | High |
| Aamir et al. 2015 | | ● | ● | ● | ● | | ● | ● | ● | High |
| Abdallah et al. 2015 | | ● | ● | ● | ● | | ● | ● | ● | High |
| Abdelmegeed et al. 2015 | | ● | ● | ● | ● | | ● | ● | ● | High |
| Abou Shady et al. 2015 | | ● | ● | ● | ● | | ● | ● | ● | High |
| Abujnah et al. 2015 | | ● | ● | ● | ● | | ● | ● | ● | High |

Summary of the assessment of risk of bias *(cont.)*

| **Cross-sectional studies** | **Selection** | | | | **Comparability** | | **Outcome** | |  |
| --- | --- | --- | --- | --- | --- | --- | --- | --- | --- |
|  | Sample representative of target population | Exposure measurement valid and reliable | Outcome measurement valid and reliable | Adequate control for confounding | | Statistical methods described and appropriate | Missing data taken into account | Sources of bias explicitely mentioned |  |
| Al-Ahmadey 2015 | ● | ● | ● | ● | | ● | ● | ● | High |
| Alaklobi et al. 2015 | ● | ● | ● | ● | | ● | ● | ● | High |
| Al Charrakh et al. 2015 | ● | ● | ● | ● | | ● | ● | ● | High |
| Al-Damoshi et al. 2015 | ● | ● | ● | ● | | ● | ● | ● | High |
| Alharbi et al. 2015 | ● | ● | ● | ● | | ● | ● | ● | High |
| Al-Humaidan et al. 2015 | ● | ● | ● | ● | | ● | ● | ● | Moderate |
| Aljanaby et al. 2015 | ● | ● | ● | ● | | ● | ● | ● | High |
| Aljindan et al. 2015 | ● | ● | ● | ● | | ● | ● | ● | High |
| Al-Khafaji et al. 2015 | ● | ● | ● | ● | | ● | ● | ● | High |
| Alsultan 2015 | ● | ● | ● | ● | | ● | ● | ● | High |
| Barguigua et al. 2015 | ● | ● | ● | ● | | ● | ● | ● | High |
| Bassyouni et al. 2015 | ● | ● | ● | ● | | ● | ● | ● | High |
| Batarseh et al. 2015 | ● | ● | ● | ● | | ● | ● | ● | High |
| Eida et al. 2015 | ● | ● | ● | ● | | ● | ● | ● | Moderate |
| Elabd et al. 2015 | ● | ● | ● | ● | | ● | ● | ● | High |
| El-Kazzaz et al. 2015 | ● | ● | ● | ● | | ● | ● | ● | High |
| Elkersh et al. 2015 | ● | ● | ● | ● | | ● | ● | ● | Moderate |
| El-Nawawy et al. 2015 | ● | ● | ● | ● | | ● | ● | ● | High |
| El-Sokkary et al. 2015 | ● | ● | ● | ● | | ● | ● | ● | High |
| Hussein et al 2015 | ● | ● | ● | ● | | ● | ● | ● | Moderate |
| Kilkal et al. 2015 | ● | ● | ● | ● | | ● | ● | ● | High |
| Lopes et al. 2015 | ● | ● | ● | ● | | ● | ● | ● | High |
| Mahmoud et al. 2015 | ● | ● | ● | ● | | ● | ● | ● | High |
| Mohamed et al. 2015 | ● | ● | ● | ● | | ● | ● | ● | High |
| Moutachakkir et al. 2015 | ● | ● | ● | ● | | ● | ● | ● | Moderate |

| Summary of the assessment of risk of bias *(cont.)* | | | | | | | | |  | |  |
| --- | --- | --- | --- | --- | --- | --- | --- | --- | --- | --- | --- |
| **Cross-sectional studies** | **Selection** | | | | | | **Comparability** | | **Outcome** | |  |
|  | Sample representative of target population | | | Exposure measurement valid and reliable | Outcome measurement valid and reliable | Adequate control for confounding | | Statistical methods described and appropriate | Missing data taken into account | Sources of bias explicitely mentioned |  |
| Rafei et al. 2015 | | ● | | ● | ● | ● | | ● | ● | ● | Moderate |
| Zakai 2015 | | ● | | ● | ● | ● | | ● | ● | ● | High |
| Zaki et al. 2015 | | ● | | ● | ● | ● | | ● | ● | ● | High |
| Zorgani et al. 2015 | | ● | | ● | ● | ● | | ● | ● | ● | High |
| Abdel-Moaty et al. 2016 | | ● | | ● | ● | ● | | ● | ● | ● | High |
| Al-Atrouni et al. 2016 | | ● | | ● | ● | ● | | ● | ● | ● | High |
| Al-Bshabshe et al. 2016 | | ● | | ● | ● | ● | | ● | ● | ● | High |
| Al Yousef, Younis et al. 2016 | | ● | | ● | ● | ● | | ● | ● | ● | High |
| Al Yousef 2016 | | ● | | ● | ● | ● | | ● | ● | ● | High |
| Al-Dahhan et al. 2016 | | ● | | ● | ● | ● | | ● | ● | ● | High |
| Al-Harmoosh et al. 2016 | | ● | | ● | ● | ● | | ● | ● | ● | High |
| Alhussaini 2016 | | ● | | ● | ● | ● | | ● | ● | ● | Moderate |
| Aljanaby et al. 2016 | | ● | | ● | ● | ● | | ● | ● | ● | High |
| Almasri et al. 2016 | | ● | | ● | ● | ● | | ● | ● | ● | High |
| Al-Mayahie et al. 2016 | | ● | | ● | ● | ● | | ● | ● | ● | High |
| Al-Otaibi et al. 2016 | | ● | | ● | ● | ● | | ● | ● | ● | High |
| Alzahrani et al. 2016 | | ● | | ● | ● | ● | | ● | ● | ● | High |
| Aziz et al. 2016 | | ● | | ● | ● | ● | | ● | ● | ● | High |
| Badran et al. 2016 | | ● | | ● | ● | ● | | ● | ● | ● | High |
| Bahy et al. 2016 | | ● | | ● | ● | ● | | ● | ● | ● | High |
| Barakat et al. 2016 | | ● | | ● | ● | ● | | ● | ● | ● | High |
| Bin Mohanna et al. 2016 | | ● | | ● | ● | ● | | ● | ● | ● | High |
| Chamoun et al. 2016 | | ● | | ● | ● | ● | | ● | ● | ● | Moderate |
| Dahdouh et al. 2016 | | ● | | ● | ● | ● | | ● | ● | ● | High |
| Elbargisi et al. 2016 | | ● | | ● | ● | ● | | ● | ● | ● | High |
| Summary of the assessment of risk of bias *(cont.)* | | | | | | | | |  | |  |
| **Cross-sectional studies** | **Selection** | | | | | | **Comparability** | | **Outcome** | |  |
|  | Sample representative of target population | | | Exposure measurement valid and reliable | Outcome measurement valid and reliable | Adequate control for confounding | | Statistical methods described and appropriate | Missing data taken into account | Sources of bias explicitely mentioned |  |
| Ghaima et al. 2016 | ● | | | ● | ● | ● | | ● | ● | ● | High |
| Jamsheer et al. 2016 | ● | | | ● | ● | ● | | ● | ● | ● | High |
| Omran et al. 2016 | ● | | | ● | ● | ● | | ● | ● | ● | High |
| Saleem et al. 2016 | ● | | | ● | ● | ● | | ● | ● | ● | High |
| Sewify et al. 2016 | ● | | | ● | ● | ● | | ● | ● | ● | High |
| Swedan et al. 2016 | ● | | | ● | ● | ● | | ● | ● | ● | Moderate |
| Abdelhamid 2017 | ● | | | ● | ● | ● | | ● | ● | ● | High |
| Abdelkader et al. 2017 | ● | | | ● | ● | ● | | ● | ● | ● | High |
| Alam 2017 | ● | | | ● | ● | ● | | ● | ● | ● | High |
| Alavudeen et al. 2017 | ● | | | ● | ● | ● | | ● | ● | ● | High |
| Aljanaby et al. 2017 | ● | | | ● | ● | ● | | ● | ● | ● | High |
| Alkasaby et al. 2017 | ● | | | ● | ● | ● | | ● | ● | ● | High |
| Alkasabi, Zaki et al. 2017 | ● | | | ● | ● | ● | | ● | ● | ● | High |
| Aziz et al. 2017 | ● | | | ● | ● | ● | | ● | ● | ● | High |
| Baaity et al. 2017 | ● | | | ● | ● | ● | | ● | ● | ● | High |
| Badawi et al. 2017 | ● | | | ● | ● | ● | | ● | ● | ● | High |
| Benaicha et al. 2017 | ● | | | ● | ● | ● | | ● | ● | ● | High |
| El-Aila et al. 2017 | ● | | | ● | ● | ● | | ● | ● | ● | Moderate |
| El-Badawi et al. 2017 | ● | | | ● | ● | ● | | ● | ● | ● | High |
| El-Gamasy et al. 2017 | ● | | | ● | ● | ● | | ● | ● | ● | Moderate |
| Elnasasra et al. 2017 | ● | | | ● | ● | ● | | ● | ● | ● | Moderate |
| Elshabrawy et al. 2017 | ● | | | ● | ● | ● | | ● | ● | ● | Moderate |
| Fadlallah et al. 2017 | ● | | | ● | ● | ● | | ● | ● | ● | High |
| Hashem et al. 2017 | ● | | | ● | ● | ● | | ● | ● | ● | High |
| Hussein 2017 | ● | | | ● | ● | ● | | ● | ● | ● | High |
| Summary of the assessment of risk of bias *(cont.)* | | | | | | | | |  | |  |
| **Cross-sectional studies** | **Selection** | | | | | | **Comparability** | | **Outcome** | |  |
|  | Sample representative of target population | | | Exposure measurement valid and reliable | Outcome measurement valid and reliable | Adequate control for confounding | | Statistical methods described and appropriate | Missing data taken into account | Sources of bias explicitely mentioned |  |
| Ktari et al. 2017 | | | ● | ● | ● | ● | | ● | ● | ● | Moderate |
| Lachhab et al. 2017 | | | ● | ● | ● | ● | | ● | ● | ● | High |
| Mashwal et al. 2017 | | | ● | ● | ● | ● | | ● | ● | ● | High |
| Natoubi et al. 2017 | | | ● | ● | ● | ● | | ● | ● | ● | Moderate |
| Saadi et al. 2017 | | | ● | ● | ● | ● | | ● | ● | ● | High |
| Soudeidah et al. 2017 | | | ● | ● | ● | ● | | ● | ● | ● | High |
| Taha 2017 | | | ● | ● | ● | ● | | ● | ● | ● | High |
| Zorgani, Almagatef et al. 2017 | | | ● | ● | ● | ● | | ● | ● | ● | High |
| Zorgani, Daw et al. 2017 | | | ● | ● | ● | ● | | ● | ● | ● | High |
| Aljanaby et al. 2018 | | | ● | ● | ● | ● | | ● | ● | ● | High |
| Al-Tamimi et al. 2018 | | | ● | ● | ● | ● | | ● | ● | ● | Moderate |
| Balkhi et al. 2018 | | | ● | ● | ● | ● | | ● | ● | ● | High |
| Gawad et al. 2018 | | | ● | ● | ● | ● | | ● | ● | ● | High |
| Hassan et al. 2018 | | | ● | ● | ● | ● | | ● | ● | ● | Moderate |
| Nairoukh et al. 2018 | | | ● | ● | ● | ● | | ● | ● | ● | High |
| Senok et al. 2018 | | | ● | ● | ● | ● | | ● | ● | ● | High |
| Soudeidah et al. 2018 | | | ● | ● | ● | ● | | ● | ● | ● | High |
| Tektook 2018 | | | ● | ● | ● | ● | | ● | ● | ● | High |
| Tohamy et al. 2018 | | | ● | ● | ● | ● | | ● | ● | ● | Moderate |
